# Cases of Tetanus; in Continuation

**Published:** 1809-11

**Authors:** John Howship

**Affiliations:** Surgeon


### Cases of Tetanus; in Continuation; by John Howship, Surgeon.

### *A rare Case of complete Tetanus, from which the Patient recovered.*

THE following case of Tetanus seems to possess an importance upon several accounts. 1st. It was the consequence of a wound, and therefore might be considered to have have arisen under the most unfavourable circumstances; notwithstanding which, the patient, after very severe sufferings, eventually recovered. 2dly, The progress of the disorder was in this case attended with certain phenomena, varying considerably from the most usual forms of the complaint. 3dly, The bowels were more disturbed, and required more attention, in this case, than happens in most examples of this disease.

In the cases formerly related, no particular remarks upon the state of the bowels were noted, because it happened that little or no disorder, in this respect, was in any of them observed. The tendency was generally to a costive habit of body, which was very readily corrected from time to time, either by the ol. Ricin. or when that was not convenient, by a laxative enema; and this tendency was readily explained, either by the previous establishment of the mercurial diathesis, or by the interruption of the regular supplies to the stomach, incident to this complaint.

J. Milwood, a young man of very robust habit of body, private in the 13th Regiment of Foot, was in garrison with the Regiment at Gibraltar. On the 6th of January, 1805, while working at his trade, which was that of a carpenter, he received a wound from his hand-saw upon the side of the great toe; some portion both of nail and integuments were torn away by the accident, so as almost to expose the bone.

For a few days nothing very material occurred; the toe was simply dressed; a small slough separated, and left a clean granulating surface. For the first week there was an offensive smell about the wound, so remarkable, as to suggest the probability of an exfoliation being thrown off from the bone; nothing of this kind, however, took place then, or at any subsequent period.

Upon the 10th day, he first complained that he felt a stiffness and odd sort of uneasiness about his jaws, with difficulty in opening his mouth. This had increased till he was unable to separate his jaws, even in the least degree. Added to this, he had frequent pains at the pit of the stomach, which he called cramps in his heart. Upon bending his limbs, so as to bring the extremities of the muscles nearer together than ordinary, he found that he was instantly seized with cramps in the parts contracted. At this time, ætherial and opiate draughts were given frequently.

The same treatment, with little variation, was continued to the 14th day after the accident, at which time, as his complaints complaints were evidently gaining ground, he was visited by Dr. Fellowes, physician to the forces. In addition to his former medicines, it was now directed that the ol. camphorat. c. tinct. opii. should be used as an embrocation, frequently and well rubbed in upon the angles of the jaws, and about the sides of the face and throat. He was also to be immediately got into the warm bath.

The bath was prepared, and the man had been immersed some minutes, when upon an unlucky attempt to turn himself, a very violent spasm came on, seizing upon the muscles of respiration, as well as those about the jaws and throat, attended with a peculiar sound not easily described. In a few minutes he became easier again, and had no returns of the spasm during the fifteen minutes she remained in the bath. When well dried and laid in blankets, the toe was dressed with the mildest applications.

When undressed for the bath, his shirt, and the flannel waistcoat within it, might literally have been wrung out, so violent and profuse was the perspiration in which he then was, and had continually been, for several days and nights past.

The pulse had been small and irregularly intermittent; the intermission sometimes following the third, at others the sixth, the ninth, and even the thirteenth pulsation. After being composed in bed and made warm, he found himself much better, "fresher," and easier than before, while he lay still. The pulse also was much improved, if its being softer and fuller was a favourable change, for the intermission still continued.

Any attempt to open his mouth brought on the spasm. The same effect constantly arose, upon his drawing in his breath suddenly, or with an effort. The camphorated frictions were continued with little interruption during the whole of this afternoon and evening. In the evening he believed, and said he certainly was better, and that when the pains were away, he found his jaws not so closely held together as they had been in the morning.

An enema, containing a proportion of the gum. fœtid. was thrown up about eight o'clock.

15th day. The injection had procured one motion only. The patient was not in pain in any part of his body while he continued to lay at rest. The jaws as closely fixed as ever. In the forenoon the camphorated frictions were repeated, which he said gave him both ease and comfort. He complained of the nape of his neck being remarkably sore, and tender when rubbed, the probable consequence of the the violence of the spasmodic action which had more than once taken place in this direction.

He was ordered to take a glass of good Madeira frequently; and this, perhaps, assisted in keeping up the continual and profuse sweating, that still continued to wet through his linen and bedding, notwithstanding they were frequently changed.

A laxative enema was thrown up this evening, and a draught ordered for bed-time, with mist. camphorat. et tinct. opii. During several hours of this evening he was certainly much better, and remained completely free from any degree of cramp, or pain.

About eleven in the night he was suddenly seized with a violent and universal spasm throughout his whole frame. Every part whatever became fixed, rigid, and immoveable. This attack was of so decided a nature, that the smallest and the largest of the muscles subservient to volition were equally and absolutely governed by its force.

The body and limbs were stiff and motionless. The eyes fixed and vacant. Fortunately, however, the fit did not continue longer than a minute, during which period the respiration was discovered to be continued, though perceptible only by the condensation of the halities, when received upon the surface of a mirror.

It is of interest to remark, that while in this singular state of restraint, the man was sensible, and said afterwards that he could see and hear perfectly. According to his own expression, he felt as if every part of him was fast screwed up in a vice. That he could not for his life have moved either finger or toe, and believed that he was dying.

Within half an hour after this attack, he was unexpectedly disturbed by a commencing diarrhœa, which occasioned him to use the bed pan no less than fourteen times during the night. The stools were little else than a thin offensive fluid matter.

As to the toe, from which it is reasonable to believe all the mischief arose, it was dressed for some time with lint dipt in the sp. vin. rect. Then with tinct. opii, and afterward with the fermenting poultice; but no application to it produced either pain in the part itself, nor the least alleviation to the general sufferings of the patient.

On this day, a preference was given to a warm emollient poultice, laid over a slip of lint, spread with the ung. e sabinæ. This application was proposed with a view to excite greater activity in the wounded part.

16th day, He was much better, not having been disturbed turbed by any accession of spasm during the night. About four in the morning, he was seized with a fit, which he described to have been, as if a man had taken him by the nape of his neck, and held him down in bed. The sinews of his neck were gathered up, as if catched in a fist. This lasted about a minute and a half. After his release he soon fell into a refreshing sleep. About five he had a thin but natural stool. In the fore part of the day, he was again well rubbed with the embrocation, and took the camphorated opiate draught. He said he was pretty well, but that he was cramped as soon as he either attempted to move, or to speak. The pulse was at this time 80; intermitting as formerly, at varying numbers of pulsation.

About half past seven, he was attacked again in the same manner as during the earlier part of the morning, complaining that besides the pains in his neck and jaws, his breast was greatly oppressed, as well as the fore part of his belly.

At nine this morning a draught, with forty drops of the tincture of opium were given, and the sore dressed with dry lint, covered by a warm poultice; an enema containing the fœtid gum was injected; the embrocation also was continued; thirty drops of the tincture of opium to be given every three hours. The jaws were nearly closed, but not so perfectly at this time, as to prevent the introduction of spoon meat, from which circumstance he was able to take sufficient nourishment to preserve and even improve his strength.

The day passed in tolerable ease; at eleven in the evening, however, he was unexpectedly seized with the spasms, which drew up and doubled all his limbs. The fit was not of longer continuance than half a minute, during which time he moaned, and muttered out repeatedly his desire to Heaven, that he might be taken away, and released from his sufferings. Upon being questioned as to his feelings, he said, that he could open his eyes, but that his limbs and body were fixed, as if held fast by the flesh.

He was again seized about one in the morning with the same description of attack, only in an aggravated degree of severity.

17th day passed without any return of the spasm, or particular pain.

18th day. At half past seven in the morning a fit came on, which kept the poor creature in torments, severe beyond description, for about two minutes. This was attended with moaning and great pain; the limbs being rigid gid and stiff; the pain was not referred to any particular part; but he said he was all drawn up together, as in a vice.

About ten in the morning he again fell into the same deplorable state, and at this time the pulse was very inconstant, the intermission being sometimes longer than the full time of a pulsation. The sweating, which has been astonishingly great from the first, continues to be as violent and profuse as ever. Forty drops of the tincture of opium, and half a drachm of the spt. æther, nit. comp. were with difficulty swallowed.

At eleven in the forenoon sapon. Castil. ℥ß. in solution with warm water, was injected; this however did not operate. At four in the afternoon he had a fit, which affected the whole of his frame; but it came on so instantaneously, that he was not able to give any notice of his situation to others, though in the latter moments of its duration, he uttered the most piteous moans, and soon after held up his hands. This fit continued upon him longer than any of his former attacks, it lasted nearly a quarter of an hour.

He passed the following night very badly; was very restless; frequently moaning, and said that he found himself very weak, and completely exhausted.

19th day. At two this morning he had another fit, exactly similar to those he had before endured; it lasted for three or four minutes only; at six he was again attacked, and began to moan; he said, as if he was in a press, where all his flesh was squeezed and drawn together. He was able to utter some words during the attack, although for the most part, the acuteness of his sufferings prevented him from making himself understood. This fit gradually subsided, leaving him in about three minutes after its onset.

He said that now the complaint seized him with more particular severity in the inside of the left thigh, and the inner part of the right arm, and that it gathered up in these parts first. He thought that if these parts were well rubbed with the embrocation he might be quite cured. He stated, that although the pain began in these parts, yet, before the fit left him, it was nearly equally severe in every other part of his body.

At nine this morning the pulse was small, beating 100. It was so nearly regular, that as to time the pulsations were perfectly equal, though now and then there was a perceptible faultering, so that perhaps it might be said to be a remitting, but no longer an intermitting pulse.

While the last note was taking, a medical friend called in in to see the patient, and upon examining the pulse, found an intermission every fifth stroke; knowing that just before it had been perfectly regular, the wrist was again examined, and it was ascertained, that now, every three pulsations were regular, but the fourth remained unstruck. In five minutes after it was again examined, when the intermission had again shifted, being now felt at every sixth stroke.

It presently changed again to the fifth, and afterwards to the seventh pulsation, at which periods the intermission was alternately found for some time to rest.

The sufferings of this patient were not much relieved by the plan of treatment he was upon, although he seemed, and believed himself to be somewhat better; when, upon the 22d day, it was determined to give a trial to the power of mercury in the removal of his complaints. This system was commenced by rubbing in ʒß. of the unguent. hydrarg. directly upon the spine. This was repeated in the evening; and the frictions were persevered in for several days, during which, the man continued to remain in the state above described.

27th day. The mercurial frictions were continued, and he now certainly suffers less from the violence of the fits; besides which, they are at length less frequent than they were. The most violent fit that he lately had had, was this morning, it lasted four minutes: he had felt several others during the night, but they were short, and productive, comparatively, of little distress.

He had had no stool for three days, in which time four laxative enemas had been injected. This morning about ten o'clock he took a draught, containing the ol. ricin. c. tinct. senna, which operated well. The mouth was by this time becoming moderately affected by the frictions. From this period the patient slowly, but regularly, continued to mend.

Upon the 55th day after the accident, he was very nearly quite recovered; able to get up and walk about. On enquiring of him as to his own opinion of the comparative benefit derived from the remedies employed, it appears that he always experienced great relief from the embrocation; he said, it always gave the parts about the neck and jaws a suppleness they had not before. As to the frictions of mercury upon his back, according to his explanation, they did him good, by bringing away the phlegm from his throat; and he came to this at last, that the moment the spitting commenced, the complaint began to to lessen, and from this time he constantly found that he gathered strength and spirits, till he eventually completely recovered, and was discharged from the hospital.

*Hill Street, Hanover Square,*
*October* 14, 1804.

( To be continued. )

